# Two Frontlines, One Diagnosis: Family Medicine-Neurology Collaboration Enables Early Recognition and Treatment of Acute Inflammatory Demyelinating Polyneuropathy in a Diabetic Patient

**DOI:** 10.7759/cureus.100724

**Published:** 2026-01-04

**Authors:** Charlie Vidal, Valeria Lopez Martinez, Giancarlo Gierbolini González, Judyanis Santiago Delgado

**Affiliations:** 1 Medicine, Manati Medical Center, Manati, PRI; 2 Family Medicine, Manati Medical Center, Manati, PRI; 3 Neurology, Manati Medical Center, Manati, PRI

**Keywords:** aidp, diabetic neuropathy, family medicine education, guillain-barré syndrome, interdisciplinary care, ivig, negative inspiratory force

## Abstract

Guillain-Barré syndrome (GBS) is an acute, immune-mediated polyradiculoneuropathy that often presents with rapidly progressive, symmetrical weakness and areflexia, typically following an infectious illness. Its recognition may be delayed in patients with underlying diabetic neuropathy due to overlapping sensory symptoms. We present the case of a 36-year-old woman with type 2 diabetes mellitus and chronic distal neuropathic pain who developed acute bilateral lower-extremity paralysis after a self-limited viral illness. Cerebrospinal fluid (CSF) revealed albuminocytologic dissociation, and magnetic resonance imaging (MRI) excluded cord compression. The diagnosis of acute inflammatory demyelinating polyneuropathy (AIDP) was established, and treatment with intravenous immunoglobulin (IVIG) led to clinical improvement. This case underscores the importance of collaboration between family medicine, emergency, and neurology in the early identification and management of GBS, even in the presence of comorbid neuropathies.

## Introduction

Guillain-Barré syndrome (GBS) is the leading cause of acute flaccid paralysis worldwide, with an incidence of approximately 1-2 cases per 100,000 person-years [1,2]. It typically manifests with ascending symmetric weakness, areflexia, and varying degrees of sensory and autonomic involvement [3,4]. The acute inflammatory demyelinating polyneuropathy (AIDP) variant accounts for the majority of cases in Western countries [5]. Antecedent respiratory or gastrointestinal infections are common triggers [1,3]. Early recognition and treatment with intravenous immunoglobulin (IVIG) or plasma exchange (PLEX) significantly improve outcomes [6]. By contrast, corticosteroids have not shown benefit in randomized evidence and are not recommended [7].

In patients with diabetes mellitus, pre-existing peripheral neuropathy can obscure recognition of GBS, resulting in diagnostic delays. This case highlights the crucial role of collaboration among family medicine, emergency medicine, and neurology in differentiating between acute and chronic neuropathies, ensuring early initiation of therapy, and coordinating rehabilitation.

## Case presentation

A 36-year-old woman with type 2 diabetes mellitus, chronic distal neuropathic pain, and recently diagnosed left-sided trigeminal neuralgia presented after awakening at 04:00 a.m. on September 30, 2025, unable to move her legs. Two weeks earlier, she experienced a self-limited flu-like illness. Five days before presentation, at her initial outpatient neurology evaluation due to left-sided facial stabbing pain, the neurologist documented absent bilateral knee and ankle reflexes. Doxepin was trialed and stopped by the patient after one dose due to paresthesia.

The patient's home medications included Trijardy XR, gabapentin 600 mg daily, gemfibrozil 600 mg daily, insulin glargine, linagliptin/metformin, losartan, metformin 1000 mg twice daily, naproxen, and pioglitazone. She denied alcohol, tobacco, or illicit drug use and reported no family history of neurological disease.

Upon initial examination at the hospital, she was alert, oriented, and hemodynamically stable. Cranial nerves II-XII were intact. Upper-extremity motor strength was 5/5; lower-extremity strength was 0-1/5 bilaterally. Deep tendon reflexes were absent at the patellar, Achilles, and plantar levels. Light touch was intact above the knees with diminished pinprick at the toes. Tone was normal without spasticity or fasciculations. She was non-ambulatory.

**Figure 1:**
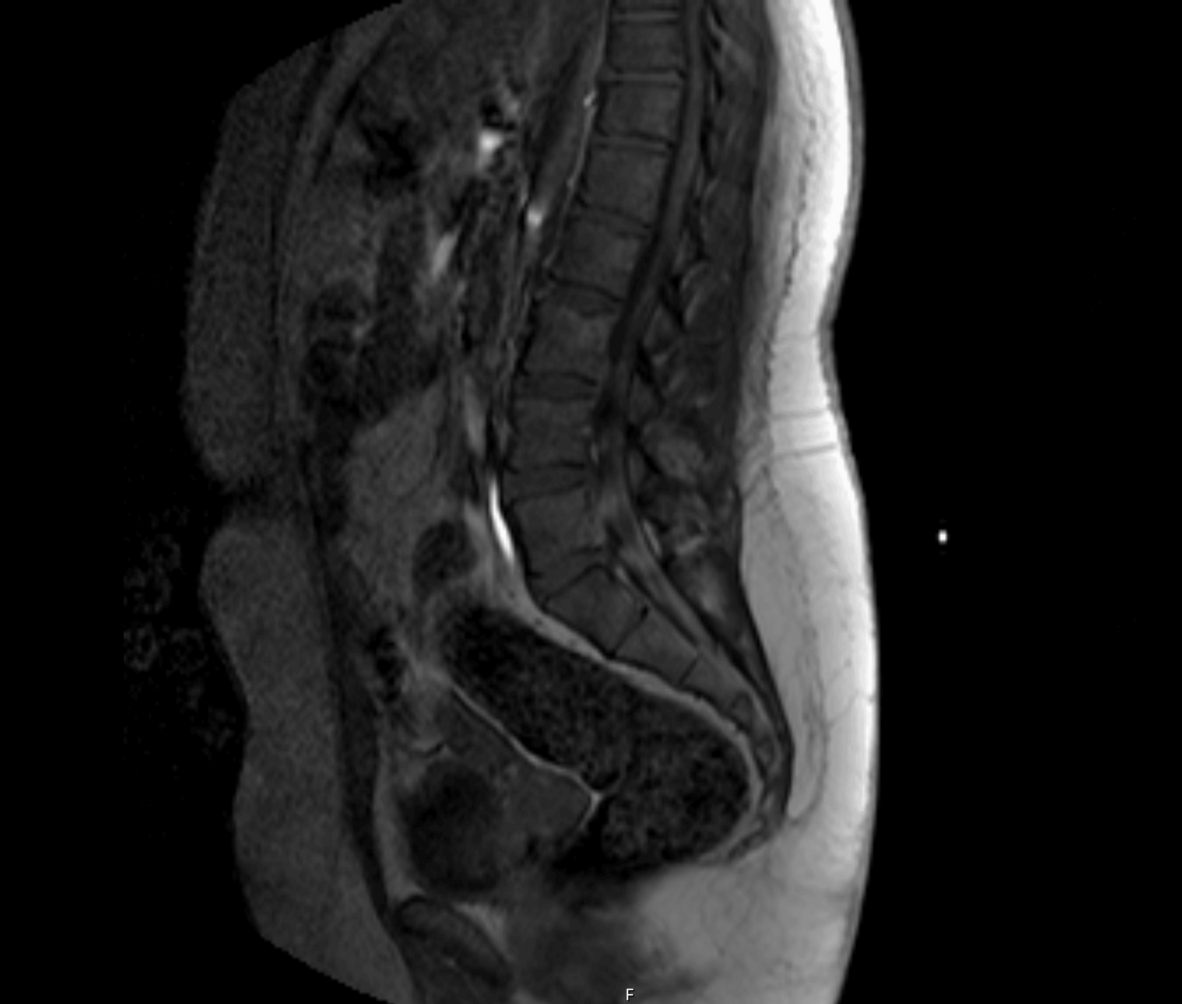
MRI of lumbar spine MRI of lumbar spine showing the normal cord signal and absence of a compressive lesion.

Respiratory assessment showed a negative inspiratory force (NIF) of -120 cm H₂O, indicating preserved inspiratory muscle strength, and oxygen saturation remained between 98% and 99% on room air. No bulbar or autonomic instability was observed. Cerebrospinal fluid (CSF) analysis revealed albuminocytologic dissociation, consistent with a demyelinating process. Magnetic resonance imaging (MRI) of the brain and entire spine showed no compressive or signal abnormalities (Figure 1).

**Table 1:** Diagnostic workup

Domain	Test/Measure	Result	Interpretation
Cerebrospinal fluid	Protein	Elevated	Albuminocytologic dissociation supports demyelinating polyradiculoneuropathy [8]
Cerebrospinal fluid	White blood cells	0 cells/mm³	Normal cellularity consistent with AIDP
Serum	Creatine kinase	Mildly elevated	Possibly immobility-related; not explanatory
Neuroimaging	MRI brain and entire spine	No mass, infarct, or compression; normal cord signal	Excludes central causes of weakness
Respiratory	Negative inspiratory force	–120 cm H₂O	Strong inspiratory effort; no respiratory muscle weakness [9]
Oxygenation	Pulse oximetry	98–99% on room air	Adequate gas exchange

Management and outcome

The patient was admitted to the intensive care unit under joint management by the family medicine and neurology teams on the day of symptom onset, following an antecedent self-limited flu-like illness two weeks earlier and an abrupt onset of complete bilateral lower-extremity weakness at approximately 4:00 a.m. Five days prior to admission, during an outpatient neurology evaluation for left-sided stabbing facial pain, absent bilateral patellar and Achilles reflexes had been documented, and a trial of doxepin was initiated but discontinued after one dose due to paresthesia. Intravenous immunoglobulin (IVIG) therapy was initiated promptly after diagnostic confirmation and administered at 0.4 g/kg/day for five consecutive days, with acetaminophen and diphenhydramine premedication and a standard rate-escalation protocol; no adverse effects occurred. This management strategy aligns with randomized controlled trials and guideline recommendations demonstrating equivalent efficacy of IVIG and plasma exchange when initiated early, with no additional benefit from combined therapy [5,6]. Corticosteroids were avoided, given moderate-quality evidence demonstrating that they do not accelerate recovery in Guillain-Barré syndrome [7].

Supportive care focused on complication prevention, stabilization, and early rehabilitation. Venous thromboembolism prophylaxis with enoxaparin and intermittent pneumatic compression devices was provided due to immobility. Glycemic control was optimized through insulin adjustments, gabapentin was continued for neuropathic pain, and physical and occupational therapy were initiated early to preserve strength, function, and prevent deconditioning. Respiratory and autonomic function were continuously monitored. Serial measurements demonstrated a sustained NIF of -120 cm H₂O and a forced vital capacity (FVC) above 20 mL/kg, both well above thresholds associated with impending respiratory failure [9]. Oxygen saturation remained at 98-99% on room air throughout hospitalization, with no bulbar weakness, arrhythmia, or blood pressure lability.

Functional status and prognosis were documented with validated tools. At admission, the Hughes Functional Grading Scale score was 4, indicating bedbound status and an inability to walk even with assistance (Table 2). Prognostic assessment was based on the modified Erasmus GBS Outcome Score (mEGOS) model [10], which incorporates age, preceding diarrhea, and initial Hughes grade as predictors of recovery. Diabetes mellitus has been reported to negatively influence short-term recovery and delay ambulation in patients with Guillain-Barré syndrome [11]. In this case, the patient’s clinical parameters predicted a favorable short-term prognosis for independent ambulation. Only factual model variables were referenced; no materials from the original publication were reproduced.

**Table 2:** Hughes Functional Grading Scale (GBS disability scale) and patient status Reproduced with attribution to Hughes RAC et al., BMJ 1978; 2:1743–1746 [12]. Used under educational fair-use for non-commercial academic purposes.

Grade	Description	Patient status
0	Healthy; no signs or symptoms	Not applicable
1	Minor symptoms or signs; able to run	Not applicable
2	Able to walk ≥10 m without assistance but unable to run	Anticipated longer-term goal
3	Able to walk ≥10 m with assistance	Anticipated rehabilitation milestone at follow-up
4	Bedbound or chairbound; unable to walk even with assistance	At admission and post-IVIG discharge status
5	Requires assisted ventilation for at least part of the day	Never required ventilation
6	Death	Not applicable

Following completion of IVIG therapy, the patient demonstrated gradual neurological improvement, with proximal lower-extremity strength increasing from 0-1/5 to 3/5. She experienced no respiratory compromise or autonomic instability and was discharged to inpatient rehabilitation with scheduled neurology follow-up.

Recent educational reviews have highlighted the importance of structured monitoring, timely referral, and interdisciplinary collaboration between family medicine and neurology in optimizing outcomes for patients with GBS [13].

## Discussion

This case demonstrates the importance of resisting diagnostic anchoring in diabetic patients with chronic neuropathy when a step-change in motor function occurs. Acute symmetric weakness with generalized areflexia and CSF albuminocytologic dissociation, in the absence of central lesions, is highly suggestive of AIDP. Collaborative management between primary care and neurology ensures early diagnosis, optimized immunotherapy, and effective rehabilitation planning.

Respiratory monitoring is essential, as approximately 25% of patients with GBS develop respiratory failure [9]. This patient’s strong inspiratory effort (NIF -120 cm H₂O) and stable FVC (>20 mL/kg) were reassuring indicators. IVIG remains first-line therapy, supported by large multicenter trials showing equivalent efficacy to PLEX when initiated within two weeks of onset [6].

The use of validated tools improved clinical communication and education. The Hughes scale provided a standardized disability measure, and mEGOS facilitated prognostic counseling [10]. Diabetes can worsen short-term recovery, further emphasizing early recognition. Collaboration between primary and specialty services ensures continuity of care, rehabilitation follow-up, and patient education. Educationally, this case highlights the need for medical trainees to recognize rapidly progressive weakness as a neurological emergency, to actively resist anchoring bias, particularly in patients with pre-existing peripheral neuropathy, to apply objective functional and prognostic scoring tools, and to coordinate interdisciplinary care between primary and specialty services, competencies that are central to contemporary clinical education frameworks.

## Conclusions

In diabetic patients, new-onset symmetric weakness should not be attributed to chronic neuropathy without evaluating for Guillain-Barré syndrome. This case highlights the impact of coordinated Family Medicine and Neurology management, guideline-based therapy, and structured monitoring using functional and prognostic tools. Early recognition and prompt IVIG therapy can prevent respiratory failure and accelerate recovery, offering key lessons for medical trainees and family medicine residents.
